# Pyridostigmine Induced Prolonged Asystole in a Patient with Myasthenia Gravis Successfully Treated with Hyoscyamine

**DOI:** 10.1155/2017/6956298

**Published:** 2017-05-14

**Authors:** Mohammad Saud Khan, Abhinav Tiwari, Zubair Khan, Himani Sharma, Mohammad Taleb, Jeffrey Hammersley

**Affiliations:** ^1^Department of Internal Medicine, University of Toledo Medical Center, Toledo, OH, USA; ^2^Department of Internal Medicine and Department of Pulmonary/Critical Care, University of Toledo Medical Center, Toledo, OH, USA

## Abstract

Reversible acetylcholinesterase inhibitors are used as first-line treatment for myasthenia gravis. They improve symptoms by increasing concentration of acetylcholine at the neuromuscular junction and stimulating nicotinic receptors. Serious bradyarrhythmias can occur from muscarinic stimulation in heart, which in rare cases may progress to asystole. These patients can initially be managed with hyoscyamine, a muscarinic antagonist. Persistence of bradyarrhythmias even after hyoscyamine treatment may warrant pacemaker placement. We present a case of 65-year-old female patient who presented with diplopia, dysphagia, and muscle weakness who was diagnosed with myasthenia gravis. She developed significant sinoatrial node block with prolonged asystole after starting treatment with pyridostigmine which was successfully treated with hyoscyamine, thus avoiding pacemaker placement.

## 1. Introduction

Myasthenia gravis (MG) is an autoimmune disorder affecting the neuromuscular junctions. It is characterized by decrease in the number of available nicotinic acetylcholine receptors at postsynaptic membrane due to presence of pathogenic autoantibodies [1, 2]. Reversible acetylcholinesterase inhibitors are used as initial treatment for MG and pyridostigmine is the most widely used drug. They improve symptoms by increasing concentration of acetylcholine at the neuromuscular junction leading to stimulation of nicotinic receptors at postsynaptic membranes [2, 3]. However, use of these drugs is associated with potential adverse effects due to stimulation of muscarinic receptors elsewhere. In heart, this results in slowing of sinoatrial (SA) rate and atrioventricular (AV) conduction [3]. Although sinus bradycardia and hypotension are common side effects of these drugs, prolonged asystole is very rare with only one case reported so far [4].

We present a case of 65-year-old female patient who presented with diplopia, dysphagia, and muscle weakness who was diagnosed with myasthenia gravis. She developed significant SA node block with prolonged asystole after starting treatment with pyridostigmine.

## 2. Case Description

A 65-year-old African American female presented with 4-month history of intermittent dysphagia, diplopia, fatigue, and muscle weakness which was more prominent in upper extremities.

Examination of cranial nerves revealed diplopia on right gaze, bilateral ptosis on sustained upward gaze, and mild lower facial muscle weakness. Motor examination showed decreased muscle strength of 4/5 in proximal and distal musculature of all extremities and neck flexors. Sensory system was intact and reflexes were normal.

In view of the above clinical presentation, MG was suspected and serological workup for MG was obtained which was positive for antibodies to acetylcholine receptor (AChR) with a titer of 14.6 nanomole/liter (0.0–0.4 nanomole/liter). Once diagnosis of MG was confirmed, she was started on pharmacological treatment with oral pyridostigmine (60 mg Q8 hours) and intravenous (IV) methylprednisolone (125 mg daily). Computerized Tomography (CT) of chest was done to exclude thymoma.

**Figure 1:**
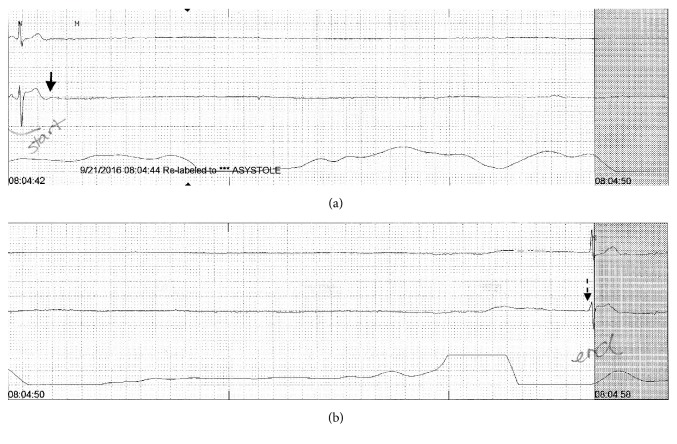
Telemetry monitor strips showing the duration of asystole with solid arrow pointing to the start of asystole and dotted arrow the end of asystole.

**Figure 2:**
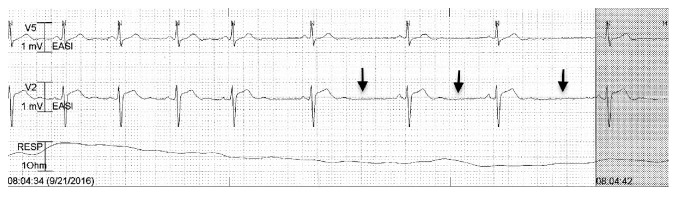
Telemetry monitor strips showing frequent sinus pauses (arrows) preceding the asystole.

On 2nd day of treatment patient developed bradycardia with heart rate ranging from 40 to 50/min and frequent sinus pauses lasting from 1 to 3 seconds (Figure 2). She then developed an episode of asystole lasting 16 seconds (Figure 1), which was picked up by telemetry. Patient was asleep during this period and reported no symptoms. The etiology of this prolonged asystole was attributed to high grade SA node block caused by pyridostigmine. The dose of pyridostigmine was decreased to 30 mg q8 hours orally; however, patient continued to have sinus pauses and bradycardia. Therefore, treatment with oral muscarinic antagonist hyoscyamine (0.25 mg q6 hours) was initiated, which resulted in improvement in bradycardia and sinus pauses. Patient did not have any known history of bradyarrhythmias or dysautonomia. She did not report any symptoms (postural dizziness, syncope, or transient weakness) concerning for primary or secondary autonomic dysfunction. On reviewing the medications, no other drug besides pyridostigmine that can cause severe bradycardia or asystole was found. She was on continuous telemetric monitoring for 72 hours after starting treatment with hyoscyamine and no further episodes of bradycardia or asystole were reported. At 1-month follow-up after discharge, patient did not complain of any symptoms of dizziness or syncope. An electrocardiogram done at the follow-up visit showed normal sinus rhythm.

## 3. Discussion

Myasthenia gravis is the most common disorder affecting the neuromuscular junctions. It is caused by antibody mediated autoimmune attack against postsynaptic nicotinic acetylcholine receptors [1, 2]. The estimated incidence of MG is around 20 to 30 per 1,000,000 per year with increased incidence with age in males and females [5, 6].

The characteristic clinical feature includes fatigable weakness of muscles with early involvement of facial and extraocular muscles [7]. Ptosis, diplopia, dysphagia, and dysarthria are common initial presentation of MG with weakness becoming more generalized and affecting the limb muscles in majority of patients in later stage of disease [2, 7]. Diagnosis of MG is clinical and suspected cases are confirmed with testing. Demonstration of autoantibodies in presence of clinical features is diagnostic of MG. Acetylcholine receptor (AChR) antibodies are most common and occur in 85% of patients [5]; however, small proportion of patients have other antibodies such as muscle specific kinase antibodies (anti-MuSK), anti-smooth muscle antibodies, and striational antibodies (anti-muscle titin and anti-ryanodine receptor) [8]. Electrophysiological studies such as repetitive nerve stimulation and single fiber EMG are also used for confirming diagnosis of MG. Tensilon test by injection of IV edrophonium and demonstration of improvement of muscle weakness is usually reserved in patients with typical clinical features and negative serological and electrophysiological testing [2].

MG is known to have cardiac involvement manifesting in different forms, ranging from asymptomatic EKG changes to fatal arrhythmias, myocarditis, heart failure, and sudden cardiac death [9, 10]. Arrhythmia is the most common clinical manifestation of cardiac involvement in MG with increased risk of sudden cardiac death.

Guglin et al. [9] studied 108 patients with MG and found that 16% of these cases have signs of cardiac involvement (atrial fibrillation, atrioventricular heart blocks, asystole, and sudden death) which were attributed to autoimmune myocarditis. The prevalence of cardiac involvement is higher in patients with thymomas (50%) as compared to patients without thymomas (12%) [10].

Treatment of MG is with acetylcholinesterase inhibitors and immunosuppression. Plasmapheresis and IVIG treatment are used in acute myasthenia crisis to expedite recovery. Pyridostigmine, a reversible acetylcholinesterase inhibitor, improves symptoms by increasing acetylcholine concentration at neuromuscular junction and stimulating nicotinic receptors [3]. However, this beneficial effect of pyridostigmine at neuromuscular junctions is accompanied by unwanted muscarinic activity in other organs which can lead to serious side effects. In heart, augmentation of vagal nerve tone causes depression of the SA and AV nodes which can lead to fatal bradyarrhythmias. Arsura et al. [11] described 12 patients of MG from a pool of more than 1000 who suffered hypotensive episodes and syncope related to use of acetylcholinesterase inhibitors. Out of these twelve patients nine suffered from severe sinus bradycardia, junctional bradycardia, or complete AV dissociation [11]. Gehi et al. [12] in 2008 reported a case of MG who presented with syncope caused by pyridostigmine induced high grade AV block. Patient was treated with hyoscyamine resulting in reversal of AV block and thus avoiding pacemaker placement. Said et al. [4] reported first case of pyridostigmine induced high grade SA block leading to asystole for 5.9 sec in a patient with MG. The patient continued to have sinus bradycardia and pauses despite being treated with anticholinergic medications and required pacemaker placement. In our patient, the asystole due to high grade SA nodal block caused by the pyridostigmine was more prolonged and lasted for 16 sec (Figure 1). SA nodal block responded to treatment with hyoscyamine and patient did not requir pacemaker placement. To our knowledge, this is second reported case of pyridostigmine induced high grade SA node block leading to prolonged asystole.

## 4. Conclusion

This is a case of pyridostigmine induced high grade SA block with longest reported duration of asystole. Treatment of myasthenia gravis with reversible acetylcholinesterase inhibitors like pyridostigmine may be associated with potential side effects such as bradyarrhythmias and in rare cases complete asystole and sinus arrest. These patients can initially be managed with hyoscyamine, a muscarinic antagonist. Persistence of bradyarrhythmias even after hyoscyamine treatment may warrant pacemaker placement.
